# Connexin30.3 is expressed in mouse embryonic stem cells and is responsive to leukemia inhibitory factor

**DOI:** 10.1038/srep42403

**Published:** 2017-02-13

**Authors:** Mikako Saito, Yuma Asai, Keiichi Imai, Shoya Hiratoko, Kento Tanaka

**Affiliations:** 1 Department of Biotechnology and Life Science, Tokyo University of Agriculture and Technology, 2-24-16, Naka-cho, Koganei, Tokyo 184-8588, Japan

## Abstract

The expression of 19 connexin (*Cx*) isoforms was observed in the mouse embryonic stem (ES) cell line, EB3. Their expression patterns could be classified into either pluripotent state-specific, differentiating stage-specific, or non-specific *Cxs*. We focused on *Cx30.3* as typical of the first category. *Cx30.3* was pluripotent state-specific and upregulated by leukemia inhibitory factor (LIF), a specific cytokine that maintains the pluripotent state of ES cell, via a Jak signaling pathway. Cx30.3 protein was localized to both the cell membrane and cytosol. The dynamic movement of Cx30.3 in the cell membrane was suggested by the imaging analysis by means of overexpressed Cx30.3-EGFP fusion protein. The cytosolic portion was postulated to be a ready-to-use Cx pool. The *Cx30.3* expression level in ES cell colonies dramatically decreased immediately after their separation into single cells. It was suggested that mRNA for *Cx30.3* and Cx30.3 protein might be decomposed more rapidly than mRNA for Cx43 and Cx43 protein, respectively. These indicate possible involvement of Cx30.3 in the rapid formation and/or decomposition of gap junctions; implying a functional relay between Cx30.3 and other systems such as adhesion proteins.

Animal cell systems generally conduct intercellular communication via cell–cell contact. Multiple cellular functions exist for (1) the detection of physical contact, (2) molecular coupling by cell membrane permeable molecules, and (3) endo/exocytosis. This topic is part of basic biology and is also of practical significance since it focuses on various, specific diseases. To date, a large number of studies on intercellular communication via cell–cell contact have been performed, which mostly speculate on the underlying molecular mechanisms involved. However, various questions remain, especially concerning functional relays supposedly existing between the three cellular processes described above. More recently, based on the methodological innovation of viable, single-cell analysis, novel conceptual subjects such as cell–cell competition^1^,^2^ and spatiotemporal synchronization^3^,^4^ have been emphasized.

Herein, we have focused on gap junction intercellular communication as a predominant feature of the second category mentioned above. A gap junction is composed of channel-forming transmembrane proteins such as connexins^5^,^6^,^7^ and pannexins^8^,^9^. There are 21 and 20 connexin (*Cx*) isoforms in human and mouse genomes, respectively^10^,^11^,^12^. A large number of studies have revealed that the expression profiles of *Cx* isoforms and their mutants vary in different species, tissues, growth stages, physiological states, and diseases^13^,^14^,^15^,^16^,^17^. Based on the analysis of predominant isoforms, such as *Cx43* and *Cx26*, the gap junction life cycle has been well explained^18^,^19^,^20^.

The specificity of function for each Cx isoform, however, is not yet fully understood. In particular, a potential mechanism for the direct detection of cell–cell contact has never been described, possibly because this would be attributed to adhesion proteins such as cadherins and integrins^21^,^22^,^23^,^24^. Curiously, however, there are few reports on the interaction between adhesion proteins and gap junctions. One report described a positive correlation between the expression of *Cxs* and the expression of adhesion proteins in colorectal cancer cells^25^. In contrast, another report described how epithelium cadherin-mediated cell–cell adhesion alone was neither essential nor sufficient to initiate *de novo* gap junction assembly in human squamous carcinoma cells^26^. Therefore, it is still unclear whether gap junctions are regulated by adhesion proteins or vice versa. We intended to find a *Cx* isoform that was sensitive to cell–cell contact events because such an isoform may be linked to the function of category (1) described above.

The functional roles of Cx proteins are not limited to the formation of gap junctions, but also extend to their involvement in cell proliferation and differentiation^6^,^27^,^28^. For example, the endocytosis of gap junctions comprising Cx43 was induced by epidermal growth factor (EGF)^20^. After internalization, Cx43 was phosphorylated by mitogen-activated protein kinase (MAPK) and protein kinase C (PKC) to promote cell migration and proliferation^29^. This indicated a negative correlation between gap junction function and cell proliferation. On the other hand, the downregulation of *Cx43* expression by siRNA inhibited both gap junction function and cell proliferation^28^, indicating their positive correlation. Therefore, it is still questionable whether the correlation between gap junctions and cell proliferation is positive or negative.

Our strategy towards the elucidation of, so far, questionable roles of Cxs in various cellular activities was to focus on embryonic stem (ES) cells. A dramatic change from the pluripotent state to an early stage of differentiation in ES cells is of general biological significance. It is well recognized that the pluripotent state of mouse ES cells can be maintained by a specific cytokine, leukemia inhibitory factor (LIF). When LIF is removed from the medium, ES cells become differentiated. When the cells are at a pluripotent or naive state, symmetric cell division for self-renewal should predominate. In contrast, cell divisions for differentiation will be mostly asymmetric. Such a cell division type should be regulated by gap junctions.

The first step in our strategic study was the global analysis of the dynamic expression pattern of every *Cx* isoform. The expression patterns of *Cxs* can be varied by numerous factors as described above. Also, differences in *Cx* patterns according to the ES cell line studied should be expected. In fact, our preliminary results for a mouse ES cell line, EB3, differed from those of a pioneering study using a different mouse ES cell line, HM1^12^. Consequently, we have found *Cx30.3* to be responsive to LIF and also to variations in conditions for cell–cell contact.

Until now, the concept of a LIF-responsive *Cx* has never been described. It has therefore been necessary to investigate the relevance of LIF and *Cx30.3* signaling to already known pathways, such as from LIF to *Oct3/4* and *Nanog*. The LIF signal is understood to be received by gp130 and LIF receptor β at the cell membrane, and then transduced to intracellular signaling pathways such as Jak-Stat3, PI3 kinase-Akt, and MAP kinase^30^. All three pathways link to pluripotency, with factors such as *Oct3/4* and *Nanog* in common.

As for cell–cell contact conditions, we compared *Cx30.3* expression and protein localization in ES cell colonies as well as single cells. According to the gap junction life cycle, the formation of gap junctions as well as of their decomposition are regulated by cell–cell contact conditions. However, the involvement of different Cx isoforms in cell–cell contact regulation has never been described. Considering the varied expression of various *Cx* isoforms, case sensitive *Cxs* and ubiquitously expressed *Cxs* may be differently involved in the regulation of cell–cell contacts. An analysis of the spatiotemporal localization of Cx30.3 protein, its dynamic variation, and kinetic studies of its mRNA and protein half-lives will reveal unique properties of Cx30.3, with important implications for other systems relevant to cell–cell contact recognition.

## Results

### Dynamic expression patterns of *Cx* isoforms during growth stage from the pluripotent state to an early stage of differentiation

**Figure 1:**
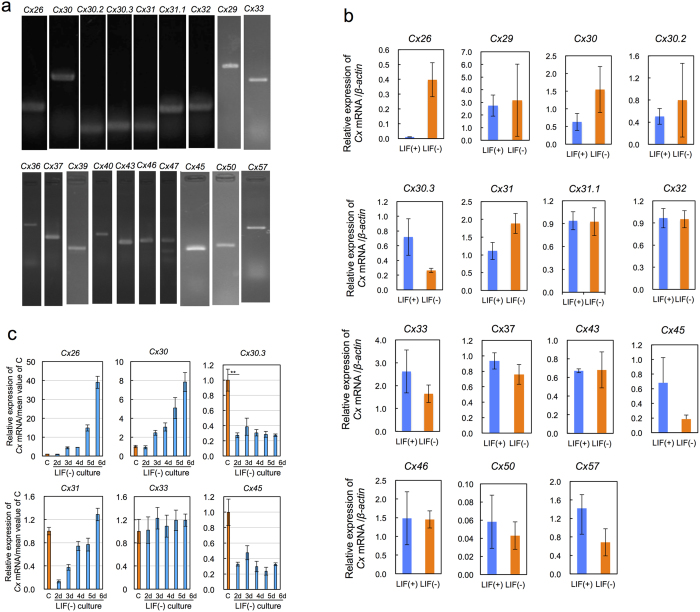
Dynamic expression of *Cx* isoforms in mouse EB3 cells. (**a**) The expression of 19 *Cx* mRNAs analyzed by RT-PCR. Refer to Table S1 for primer sets and predicted band sizes. (**b**) Changes of expression levels of *Cxs* during culture in LIF(−) medium analyzed by qRT-PCR. LIF(+): Culture in LIF(+) medium for 3 d, LIF(−): Culture in LIF(−) medium for 6 d. Refer to Table S2 for primer sets. mean ± SD for n = 3. (**c**) Typical examples of dynamic gene expression patterns analyzed by qRT-PCR. C: control, cultured in LIF(+) medium for 3 d. nd: cultured in LIF(−) medium for *n* d. mean ± SD for n = 3. Refer to Table S2 for primer sets. **: statistically significant by Student’s *t*-test p < 0.01.

Among 20 *Cx* isoforms in mouse genome, the gene expressions of 19 isoforms were detected in the pluripotent state of EB3 cells (Fig. 1a) and 15 of them showed the gene expression also in an early stage of differentiation that was defined as the stage after the culture for 6 d in the medium containing no LIF (LIF(−) medium) (Fig. 1b). The gene expression levels in both stages were same or markedly different. Ten isoforms such as *Cx29, Cx32*, and *Cx43* were the former case. The dynamic changes of the latter case plus one (*Cx33*) of the former case were analyzed by qRT-PCR (Fig. 1c). Respective genes showed three different expression patterns: (1) higher expression in the pluripotent state than in the early stage of differentiation (*Cx30.3, Cx45*), (2) lower expression in the pluripotent state than in the early stage of differentiation (*Cx26, Cx30*), or (3) constant expression throughout the time period (*Cx33*), or a decrease-then-increase mode of expression (*Cx31*). Of these 6 isoforms, we focused on *Cx30.3* because its expression behavior was thought to be predominantly associated with the pluripotent state.

### Expression of Cx30.3 as protein determined by western blot analysis

**Figure 2:**
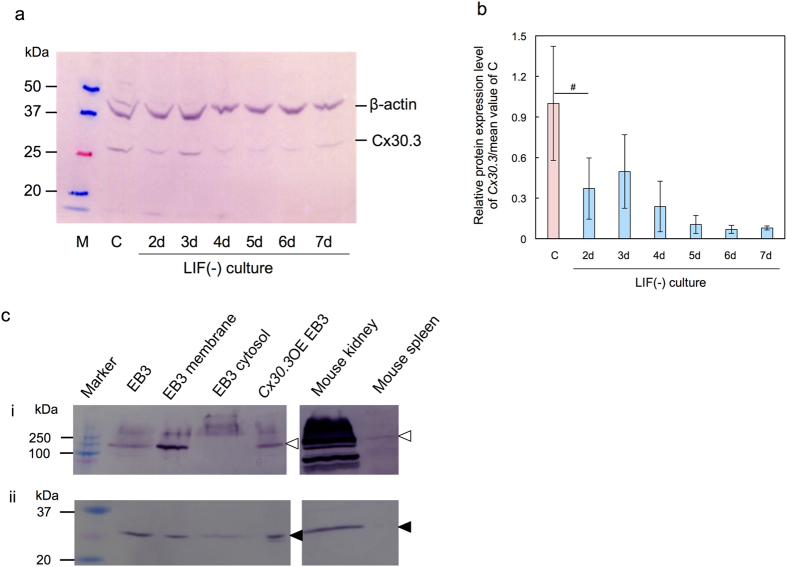
Cx30.3 protein expression detected by western blot analysis. (**a**) Growth stage dependent variation. M: marker, C: control, cultured in LIF(+) medium for 3 d. nd: cultured in LIF(−) medium for n d. (**b**) Quantified figure of the result a. mean ± SD for n = 3. Statistical significance: ^#^p < 0.1 by Student’s *t*-test. (**c**) Cell membrane/cytosol localization. EB3 membrane: the cell membrane fraction of EB3 cells, EB3 cytosol: the cytosol fraction of EB3 cells, *Cx30.3*OE EB3: *Cx30.3* overexpressing EB3 cells. i: Detection of α1 Na^+^ -K^+^ ATPase (◃), ii: Detection of Cx30.3 (◂).

Western blot analysis revealed that Cx30.3 protein was expressed when cells were in the pluripotent state (Fig. 2a,b). The quantity of Cx30.3 protein decreased during culture in LIF(−) medium for 2 d. The expression profile of Cx30.3 protein was consistent with its transcription activity profile (Fig. 1c, *Cx30.3*).

Then the test sample was fractionated by ultracentrifugation to analyze whether Cx30.3 protein was located in cell membrane or cytosol. The cytosol fraction was not contaminated with cell membrane fraction as supported by the result of α1 Na^+^ -K^+^ ATPase, a cell membrane marker. As depicted in Fig. 2c, Cx30.3 was localized not only in the membrane protein fraction but also in the cytosol fraction.

### LIF to Cx30.3 signaling pathway

According to a former ref. 30, the LIF signaling pathway involved *Jak-Stat3, PI3 kinase-Akt*, and *Grb2-MAP kinase* pathways. *Klf4* and *Tbx2* were the next downstream factors of *Stat3* and *Akt*, respectively and upregulated. *Tbx2* was also the next downstream factor of *MAP kinase*, though it was downregulated. The *Jak-Stat* pathway could be downregulated by the removal of LIF and then re-activated by the re-addition of LIF. In contrast, such a re-activation was not observed with the *PI3 kinase-Akt* pathway. Here we investigated the involvement of *Cx30.3* in these pathways using *Klf4* and *Tbx2* as specific markers of respective pathways.

**Figure 3:**
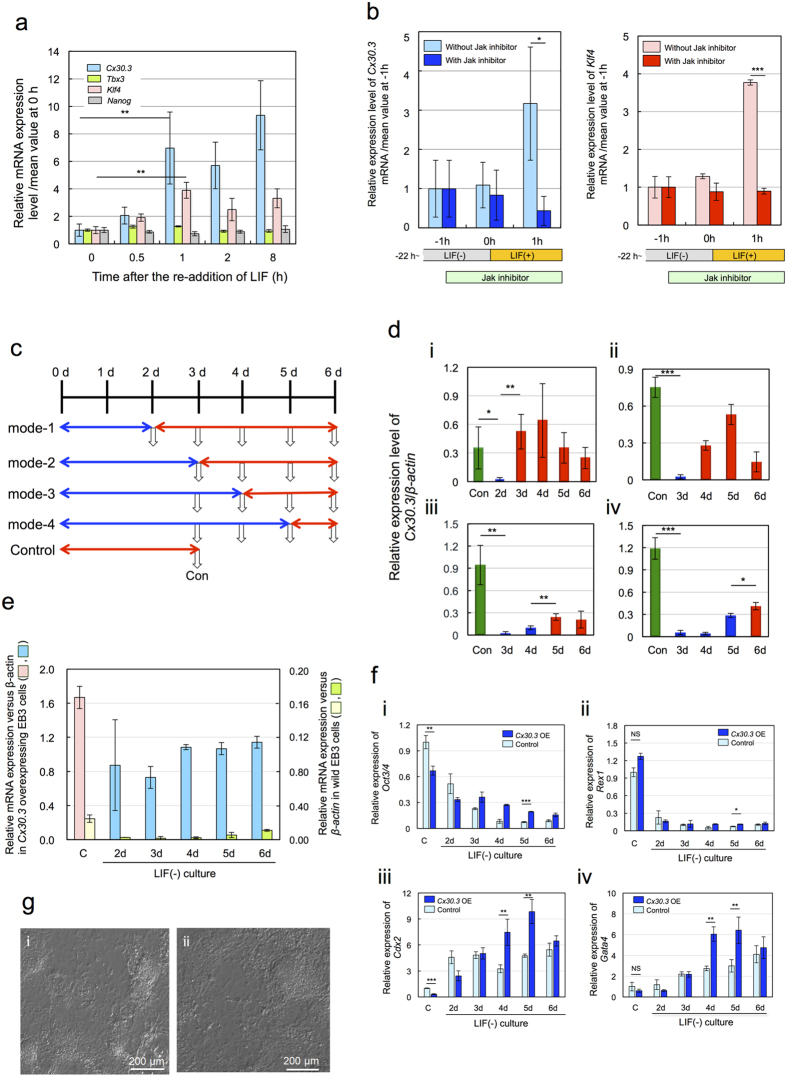
LIF-responsive expression of *Cx30.3*. (**a**) Effects of the re-addition of LIF on the expression of relevant genes. mean ± SD for n = 3. Statistical significance: **p < 0.01 by Student’s *t*-test. (**b**) Effects of Jak inhibitor on the re-activation of *Cx30.3* and *Klf4* by the re-addition of LIF. mean ± SD for n = 3. Statistical significance: *p < 0.05, ***p < 0.001 by Student’s *t*-test. (**c**) Growth stage control conditions and test sample collection. Blue line: culture in LIF(+) medium, red line: culture in LIF(−) medium, Control: culture for 3 d in LIF(+) medium. White arrows: test sample collections. Con: controls prepared and tested simultaneously with samples i, ii, iii, iv, respectively, in D. (**d**) Dynamic expression patterns of *Cx30.3* in response to LIF after the culture in LIF(−) medium for 2 d (i), 3 d (ii), 4 d (iii), and 5 d (iv). Con: control. mean ± SD for n = 3. Statistical significance: *p < 0.05, **p < 0.01, ***p < 0.001 by Student’s *t*-test. (**e**) Relative expression levels of *Cx30.3* in overexpressing EB3 cells and in wild EB3 cells versus that of *β-actin*. C: Control EB3 cells or EB3 overexpressing cells cultured in LIF(+) media, nd: Cultured in LIF(−) medium for n days. mean ± SD for n = 3. (**f**) Effects of the overexpression of *Cx30.3* on pluripotency- and differentiation-associate genes. (i) *Oct3/4*, (ii) *Rex1*, (iii) *Cdx2*, (iv) *Gata4*. mean ± SD for n = 3. Expression levels: relative to the mean value of control in LIF(+). NS: Statistically not significant by Student’s *t*-test. Statistical significance: *p < 0.005, **p < 0.01 by Students *t*-test (**g**) Effects of *Cx30.3* overexpression on the shape of cells and colonies. (i) EB3 cells in LIF(−) for 6 d, (ii) *Cx30.3* overexpressing EB3 cells in LIF(−) for 6 d.

EB3 cells were cultured in LIF(−) medium for 21 h to cease the LIF signal and then the medium was replaced by LIF(+) medium. *Cx30.3* could be re-activated by the re-addition of LIF in a more remarkably than *Klf4* (Fig. 3a). However, neither *Tbx2* nor *Nanog* could be re-activated. Then we investigated the effect of Jak inhibitor on the re-activation of *Cx30.3* and also on the re-activation of *Klf4* as a positive control. After the culture in LIF(−) medium for 21 h and then in LIF(−) medium containing Jak inhibitor for 1 h, the medium was replaced by LIF(+) medium containing Jak inhibitor. Consequently, both *Cx30.3* and *Klf4* could be re-activated without the inhibitor, while being inhibited completely with the inhibitor (Fig. 3b). Therefore, *Cx30.3* was speculated as a downstream factor branching from *Jak*. This LIF to *Cx30.3* signaling pathway, however, did not link to *Nanog*.

### Growth stage dependence of re-activation of *Cx30.3* by re-addition of LIF

Re-activation by re-addition of LIF was regarded as a characteristic property of the LIF to *Jak-Stat3* signaling pathway^30^. Then we investigated whether such a property could be maintained only in the pluripotent state or even after differentiation. Experimental protocol is shown in Fig. 3c. According to the mode-1, the result of Fig. 3d-i was obtained. During culture of EB3 cells in LIF(−) medium for 2 d, *Cx30.3* expression levels decreased to 10 times lower than that of control cells cultured in LIF(+) medium for 3 d. After the medium was subsequently replaced by LIF(+) medium, however, the *Cx30.3* expression level immediately increased to a higher level than that of the control within 1 d (Fig. 3d-i, result of “3 d”). In the mode-2, culture in LIF(−) medium was continued for 3 d and then the medium was replaced by LIF(+) medium. The *Cx30.3* expression could be re-activated and its level became rapidly 10 times higher than that of 3rd day within 1 d (Fig. 3d-ii, results of “3 d” and “4 d”). However later addition of LIF according to the mode-3 or mode-4 was not effective to the re-activation of *Cx30.3* (Fig. 3d-iii).

The empirical criteria of the culture condition for maintaining pluripotent state or initiating differentiation is as follows (personal communication). The culture in LIF(−) medium for longer than 3d is the criteria for the differentiation without reversible turning to the pluripotent state even in the LIF(+) medium, though no scientific reason of 3 d has not been clarified. According to this criteria, EB3 cells cultured in LIF(−) medium for no longer than 3 d are thought to be mostly still at the pluripotent state. The re-activation of *Cx30.3* occurred only when the cells were staying at this pluripotent state.

### Regulation of pluripotency- and differentiation- associated genes by *Cx30.3*

If the LIF to *Cx30.3* signaling pathway links to pluripotency-associated genes such as *Oct3/4* and *Rex1*, the pluripotent state may be maintained by the overexpression of *Cx30.3* alone. Then we investigated its possibility by the culture of Cx30.3 overexpressing EB3 cells in LIF(−) medium. At first we confirmed that the *Cx30.3* expression in the *Cx30.3* overexpressing EB cells could maintain its sufficiently high level throughout 6 d even in the LIF(−) medium (Fig. 3e). Under this condition, however, neither *Oct3/4* nor *Rex1* showed any remarkable response (Fig. 3f-i,ii).

On the other hand, we suspected that the *Cx30.3* overexpression might contribute to the maintenance of pluripotent state by the downregulation of differentiation-associated genes such as *Cdx2* and *Gata4*. As depicted in Fig. 3f-iii, the expression of *Cdx2* alone was suppressed slightly at pluripotent state. Both genes were upregulated rather than suppressed after the initiation of differentiation (Fig. 3f-iii,iv, at 4 d and 5 d).

In summary the effects of *Cx30.3* overexpression, if any, on the maintenance of pluripotent state were not enough to be alternative to LIF.

### Effects of *Cx30.3* overexpression on cell and colony shape

The potency of maintaining the pluripotent state can be evaluated by direct observation of the shape of cells and colonies. A *Cx30.3* overexpressing EB3 cells cultured in LIF(−) medium for 6 d became colonies with irregular shapes (Fig. 3g-i), indicating differentiated state. Therefore *Cx30.3* alone could not maintain the morphology of pluripotent state of ES cells.

### Localization of endogenous Cx30.3 and Cx43 proteins

**Figure 4:**
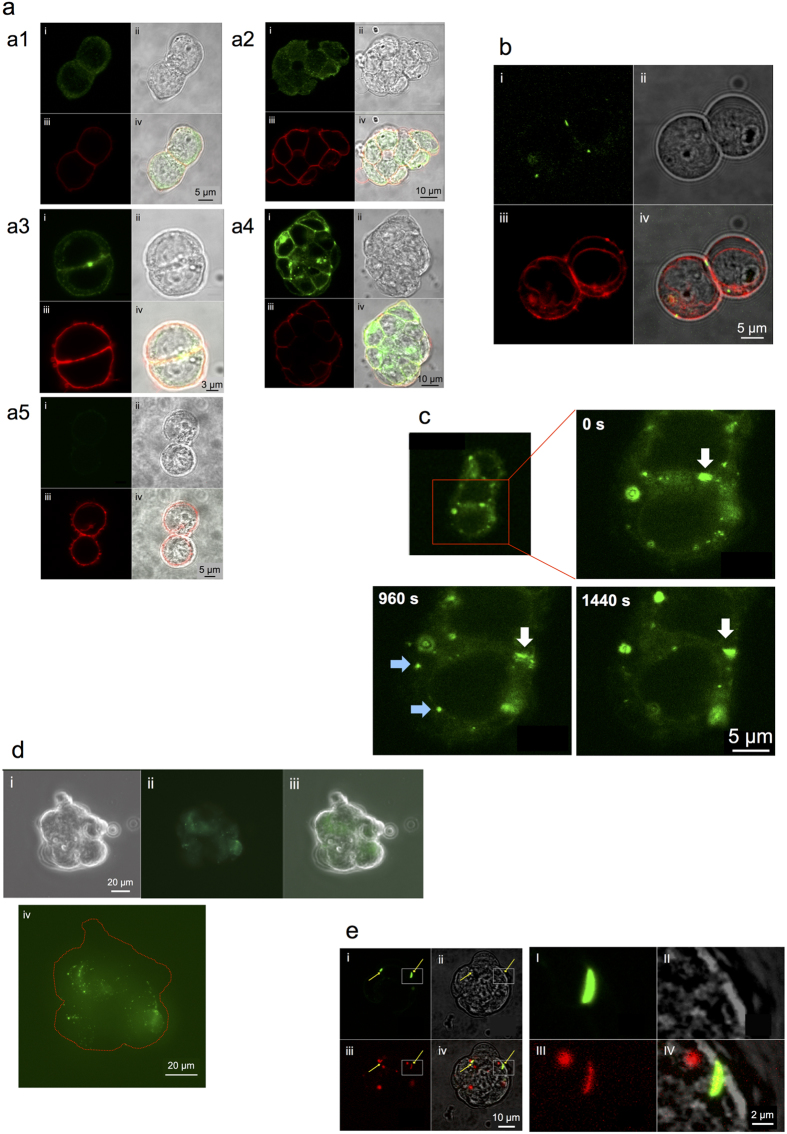
Localization of Cx30.3 protein and/or Cx43 protein. (**a**) Localization of endogenous Cx30.3 and Cx43 proteins. (a1) Cx30.3 in contacting 2 EB3 cells. (a2) Cx30.3 in an EB3 cell cluster. (a3) Cx43 in contacting 2 EB3 cells. (a4) Cx43 in an EB3 cluster. (a5) control stained with the second antibody. (i) Cx30.3 or Cx43 image, (ii) bright field image, (iii) membrane structure stained with PKH26, (iv) merge of i - iii. (**b**) Cx30.3-EGFP localization in contacting 2 EB3 cells. (i) Cx30.3-EGFP image, (ii) bright field image, (iii) membrane structure stained with PKH26, (iv) merge of i - iii. (**c**) Dynamic localization of Cx30.3-EGFP in a small colony. White arrow: gap junction plaque moving in cell-cell contact membrane region, Blue arrows: assumed hemi-channels that disappeared within 480 s ( = 1440–960). (**d**) Cx30.3-EGFP localization in a large colony of Cx30.3-EGFP overexpressing EB3 cells. (i) bright field image, (ii) Cx30.3-EGFP image, (iii) merge of i and ii, (iv) enlarged image of (ii) with the outermost peripheral line of the colony indicated in a red broken line. (**e**) Co-expression of Cx30.3-DsRed and Cx43-EGFP. (i) Cx43-EGFP image, (ii) bright field image, (iii) Cx30.3-DsRed image, (iv) merge of i - iii. (I) - (IV): enlarged images of yellow squares in i - iv, respectively.

A comparative analysis of the localization of Cx30.3 and Cx43 proteins revealed the presence of Cx30.3 protein in EB3 cells. Cx30.3 protein was localized in cell membrane as well as in cytosol near cell membrane (Fig. 4-a1). The distribution in cytosol was observable more clearly in a cluster. This suggests that a large number of Cx30.3 protein might be stored in some area of cytosol. On the other hand, Cx43 was distributed dominantly in cell membrane (Fig. 4-a3). Another noticeable point was that large fluorescent spots were observed only in Cx43 images. Such spots were speculated as gap junctional plaques. In contrast, no signal was observed in the control (Fig. 4-a5).

### Dynamic localization behavior of Cx30.3 protein

The life cycle of Cx protein is roughly composed of two processes, i.e. the incorporation in cell membrane to form gap junctions and the removal of gap junctions by internalization. The dynamic behavior of Cx30.3 in these processes were investigated.

Cx30.3 protein labelled with EGFP at C-terminal was overexpressed in EB3 cells. Intense fluorescent spots were observed at the cell-cell contact region (Fig. 4b), which were ascribed to gap junction plaques. At the same time, less intense fluorescent small spots were distributed in the cytosol. Those were thought to be hexamers of Cx30.3 on the way to cell membrane. Time-lapse measurement at every 120 s for 3480 s revealed that Cx30.3-EGFP appeared, moved, or disappeared rapidly (Fig. S1). The image data captured at 0, 960, and 1440 s are displayed in Fig. 4c. A fluorescent spot indicated by a white arrow was supposedly a gap junction plaque. This plaque moved outward about 3 μm within 960 s and then seemed to stay there for successive 480 s. During the latter period, the fluorescent intensity increased, suggesting 3 dimensional movement of the plaque in the cell-cell contact membrane. On the other hand, blue arrows indicate the appearance of new fluorescent spots that were supposedly hemi-channels. These spots disappeared within 480 s possibly by internalization and decomposition.

A more drastic disappearance of Cx30.3 hemi-channels was observed in large colonies of EB3 cells. Cx30.3-EGFP was principally distributed at cell-cell contact membrane region but no fluorescent spot was observed at the outermost region (Fig. 4d). Such a phenomenon was not observed with Cx26-EGFP overexpressing EB3 cells (unpublished data).

### Half-life of overexpressed Cx protein estimated by dynamic imaging analysis

According to a former review^31^, the half-lives of Cx proteins such as Cx43, Cx32, Cx46, and Cx26 ranged 1–5 h. The Cx half-life is influenced by the cell culture conditions. For example, the half-life of Cx43 in gap junction plaques in cultured cells of corneal endothelium increased in response to an acute stressor such as genotoxic stress^32^, suggesting a temporal stabilization of gap junctional intercellular communication under a stress condition. The stabilization remarked in this context, however, was only a small change of the half-life from 1–2 h to 3–4 h. More recently, in the relevance to skin health and hearing loss, Cx30 was found to be unusually stable with a half-life longer than 12 h^33^. On the other hand, the analysis of plaques by fluorescence recovery after photobleaching (FRAP) revealed much more rapid diffusion behavior of Cx molecules within plaque structures. Cx26 and Cx30 expressed in HeLa cells diffused within 30 s, while Cx43 remained persistently immobile for more than 2 min^34^.

Taking these into consideration, the disappearance of Cx30.3 spots (Fig. 4c-blue arrows) should reflect the internalization or decomposition and not the diffusion within the plaque. From this result, the half-life of Cx30.3 was estimated as 240 s that was much shorter than those of other isoforms (1–5 h). In this case, however, Cx30.3 spots were thought to be hemi-channels and this should be a reason why such a short half-life was observed. These suggested that the removal of gap junctions and/or hemi-channels could be promoted by a signal of the decrease or loss of cell-cell contact membrane region.

### Co-localization of overexpressed Cx30.3 and Cx43

Intracellular localization of overexpressing Cx30.3 protein in EB3 cells seemed to be different from those of other Cx isoforms such as Cx26, which was also overexpressed in EB3 cells at pluripotent state (unpublished data). In fact, Cx30.3 alone stayed mostly in cytosol in HeLa cells but its transportation to the cell membrane was promoted by the co-expression with Cx31^35^ suggesting their functional close interaction.

Therefore we suspected that Cx30.3 in cell membrane of EB3 cells should be co-localized with other isoforms. Cx43, the most predominant isoform, was thought to be a candidate for such a partner of the co-localization. Consequently the co-localization of Cx30.3-DsRed and Cx43-EGFP was detected at cell-cell contact membrane (Fig. 4e). On the other hand, circular spots of Cx30.3-DsRed were located also in cytosol (Fig. 4e-III). It was not yet analyzed whether these spots were Cx30.3 alone or co-localization with other isoforms than Cx43. Such a co-localization suggested a potential role of Cx30.3 as the pool of Cxs for ready-to-use.

### Drastic downregulation of *Cx30.3* by the dissociation of colonies into single-cells

Here we simulated a situation of drastic decrease of cell-cell contact membrane region by enzymatic dissociation of ES cell colonies into single-cells. We predicted that the expression of *Cx30.3* should decrease rapidly under this condition. Colonies of pluripotent EB3 cells were treated with trypsin to dissociate them into single-cells and applied to a cell sorter. SSEA1 (stage-specific mouse embryonic antigen) stained cells were also prepared to confirm that the collected cells were pluripotent.

**Figure 5:**
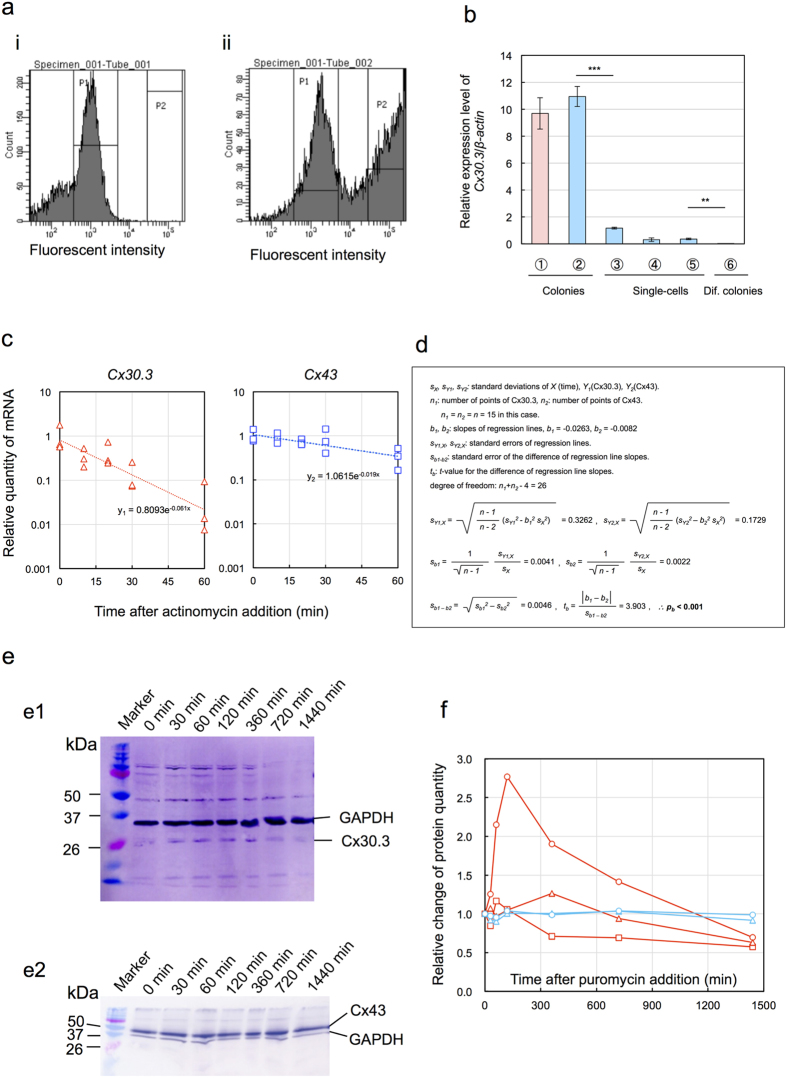
Effects of the dissociation of EB3 colonies into single-cells on *Cx30.3* expression. (**a**) Flow cytograms. (i) unstained cells, (ii) SSEA1 immunostained cells. (**b**) *Cx30.3* expression in single-cells. ①: EB3 in LIF(+) for 3 d, ②: ①+ immunostaining, ③: ① + FACS (P1 fraction in Fig. 5a-i), ④: ② + FACS (SSEA1(+), P2 fraction in Fig. 5a-ii), ⑤: ② + FACS (SSEA1(−), P1 fraction in Fig. 5a-ii), ⑥: Colonies of differentiated EB3, cultured in LIF(−) for 6 d. mean ± SD for n = 3. Statistical significance: **p < 0.01, ***p < 0.001 by Student’s *t*-test. (**c**) Decomposition of mRNA for *Cx30.3* and *Cx43*.△: Cx30.3, □: Cx43. (**d**) Calculation of t-value for the difference of regression line slopes of mRNA in C. (**e**) Dynamic changes of Cx30.3 and Cx43 protein expression revealed by western blot analysis. (e1) Cx30.3, (e2) Cx43. (**f**) Time courses of the quantity of Cx30.3 and Cx43 proteins. (red O, △, □): Cx30.3, (blue O, △): Cx43.

Unstained cells emitted auto-fluorescence (Fig. 5a-i, P1 fraction) but immunostaining with anti-SSEA1 antibody and then reacted with the second antibody labelled with a fluorescent dye generated cells with higher intensity of fluorescence (Fig. 5a-ii, P2 fraction). Cells in P1 and P2 fractions (Fig. 5a-ii) were thought to be SSEA1 negative (differentiated) cells and SSEA1 positive (pluripotent) cells, respectively. The *Cx30.3* mRNA expression intensities analyzed by qRT-PCR are depicted in Fig. 5b. The expression intensity in single-cells decreased dramatically to as low as 10% of that of colonies.

It is well recognized that LIF(+) medium can maintain EB3 cells at the pluripotent state. Under this culture condition, however, some portion of cells are somehow differentiated into a SSEA1 negative state. Under the present experimental condition, SSEA1- positive and negative cells co-existed and the Cx30.3 expression levels of both fractions were almost same. Its level was markedly lower than the control level but sufficiently higher than that of the differentiated cells cultured in the LIF(−) medium for 6 d. Therefore the marked decrease in the *Cx30.3* expression was thought to be caused by the loss of cell-cell contact membrane region and not by the loss of pluripotency.

### Half-life of mRNA for *Cx30.3*

Rapid decrease of Cx30.3 protein implied the rapid decomposition of mRNA for *Cx30.3*. Thus we compared the stability of mRNA for *Cx30.3* as well as for *Cx43* by the quantitative analysis of mRNA in the presence of a transcription inhibitor, actinomycin D. As depicted in Fig. 5c, the quantity of mRNA for *Cx30.3* decreased more rapidly than that for *Cx43*. In single logarithmic chart, regression lines were obtained by exponential function approximation as follows: *y*_*1*_ = 0.8039 exp[−0.061*x*] and *y*_*2*_ = 1.0615 exp[−0.019*x*] for *Cx30.3* and *Cx43*, respectively. Here *x* indicates time in min. From these equations, the half-lives were determined as 11.4 min ( = ln2/0.061) for *Cx30.3* and 36.5 min (=ln2/0.019) for *Cx43*, respectively. To test the significance of slope difference, the regression lines were converted to the following formulas: *Y*_*1*_ = − 0.0948–0.0265*X* for *Cx30.3* and *Y*_*2*_ = 0.0259–0.0083*X* for *Cx43*. According to the calculation steps summarized in Fig. 5d, *t*-value for the difference of regression line slopes was determined as 3.903. The degree of freedom was 26 and therefore statistical significance by Student’s *t*-test was p < 0.001.

### Decay processes of Cx30.3 and Cx43 proteins

The concentration changing process of Cx30.3 and Cx43 proteins were analyzed by western analysis (Fig. 5e) and then quantified using image J. Though the changing profiles of 3 samples of Cx30.3 were markedly varied, it was observed that Cx30.3 protein exhibited initial increase and successive decrease (Fig. 5f). It should take some time for puromycin to diffuse into cytosol and to inhibit the protein synthesis. During this lag time, Cx30.3 protein might be synthesized by means of Cx30.3 mRNA.

The successive decrease should reflect the decay process of Cx30.3 protein. In contrast, Cx43 protein showed no appreciable change of its quantity. These suggest strongly that Cx43 protein should be much more stable than Cx30.3 protein.

## Discussion

Cx30.3 is a promising candidate to elucidate the specific role of Cx in the dramatic change from the pluripotent state to an early stage of differentiation in ES cells. To date, the expression of *Cx30.3* has been observed only in differentiated functional tissues and HeLa cells, and its involvement in maintaining the pluripotent state of ES cells has not been described.

Mutations of *Cx30.1* and *Cx30.3* have been associated with erythrokeratoderma variabilis, a rare disorder of skin cornification^36^. The molecular interaction of *Cx30.3* with *Cx31* was reported to be associated with the same skin disease^35^. The heteromeric connexons of Cx30.3 and Cx31 proteins can be transported to the cell membrane to form gap junctions in HeLa cells, while Cx30.3 protein alone remains in the cytosol. Simultaneous mutations of *Cx26* and *Cx30.3* caused autosomal recessive non-syndromic hearing loss in the digenic mode of inheritance^37^. The exposure of rats to steroidal compounds caused a variation in expression levels of nine *Cx* isoforms, including *Cx30.3* in the corpus epididymis^38^. In summary, the involvement of *Cx30.3* mutations has so far been reported mostly in skin diseases and hearing loss.

A study reporting that Cx30.3 protein alone could not be transported to the cell membrane in HeLa cells^35^ strongly suggested this to be a unique property of Cx30.3. In EB3 cells, however, Cx30.3 protein was localized not only in the cytosol, but also in the cell membrane (Fig. 2c, Figs 4-a1, a2). The co-localization of Cx30.3 and Cx43 (Fig. 4e) suggested their close interaction. We suspected that Cx30.3 might be co-localized in the cell membrane also with other isoforms. In this sense, the cytosolic Cx30.3 protein might be a ready-to-use Cx pool for forming heteromeric connexons or gap junction plaques.

It was not surprising that the expression of *Cx30.3* decreased in response to a decrease of the cell–cell contact region. In fact, the expression level of ubiquitously expressed *Cx43* in EB3 colonies decreased when cells were dissociated into single cells. However its level was never lower than 50% (unpublished data). In sharp contrast, the expression level of *Cx30.3* became as low as 10%. Such a remarkable decrease strongly supports a unique role of *Cx30.3* in rapid decrease of gap junctions. The short half-life of Cx30.3 mRNA as well as of Cx30.3 protein was thought to allude to such a role for Cx30.3 by leading to the decomposition of unnecessary mRNA and protein as quickly as possible.

In conclusion, Cx30.3 is a novel isoform that has been assigned as a pluripotent state-specific isoform. It has a potential role in contributing to the quick formation and/or decomposition of gap junctions in EB3 cells. Following on from these findings, the promoter of *Cx30.3* needs to be analyzed to clarify why such rapid regulation is essential in the pluripotent state and how the cell–cell contact signal is transduced to *Cx30.3*. Clarification of the potential role of Cx30.3 as a pluripotent state-specific isoform will provide novel insights into the dynamic, functional networks required for cell–cell contact recognition.

### Online methods

#### ES cell culture

EB3, a clone of feeder-free mouse ES cells, was provided by H. Niwa (Center for Developmental Biology, RIKEN, Kobe, Japan) and cultured at 37 °C in the absence of feeder cells in medium for ES cells (ESM) on gelatin-coated dishes. ESM was composed of GMEM (Sigma, St Louis, MO, USA), 10% fetal calf serum, 1 mM sodium pyruvate, 10^−4^ M 2-mercaptoethanol, 1 × non-essential amino acids, and 1,000 U/mL of LIF. ESM containing LIF was designated as LIF(+) medium and ESM without LIF was designated as LIF(−) medium hereinafter.

#### Preparation of pluripotent and differentiated cells

Pluripotent cells were prepared by culturing EB3 cells in LIF(+) medium for 3 d. Differentiated cells were prepared by culturing pluripotent cells in LIF(−) medium for up to 6 d. The cell lineages involved in this study ranged from pluripotent ES cells, to an approximate early stage of differentiation into endodermal, ectodermal, or trophectodermal cells. A temperature of 37 °C was maintained throughout the culture period.

#### RNA isolation, reverse transcription PCR (RT-PCR), and quantitative RT-PCR (qRT-PCR)

Total RNA was prepared using ISOGEN II (Nippongene, Tokyo, Japan) according to the manufacturer’s instructions. Briefly, 70–80% confluent cells were washed with phosphate buffered saline (PBS) and suspended in 0.8 mL ISOGEN II. The prepared total RNA was then treated with DNase to obtain a purified RNA sample.

Two μg of purified total RNA was mixed with 0.5 μL of 100 ng/μL oligo (dT) primers at 70 °C for 10 min and cooled on ice for 1 min. RNA was then converted to cDNA using Super Script II reverse transcriptase (Invitrogen, Grand Island, NY, USA) according to the manufacturer’s instructions.

RT-PCR was performed in GoTaq^®^ Green Master Mix (Promega Corporation, Madison, WI, USA), according to the manufacturer’s protocol, using a Gene Amp PCR system 9700 (Applied Biosystems, Foster City, CA, USA). Amplification conditions were as follows: 94 °C for 3 min, followed by 20–40 cycles of a reaction set (94 °C denaturation for 1 min, 55–65 °C annealing for 1 min, 72 °C elongation for 2 min), with a final incubation at 72 °C for 7 min. Primers used for RT-PCR are listed in supplemental table (Table S1). PCR products were separated by agarose gel electrophoresis (100 V, 30 min) and visualized by staining with ethidium bromide.

The expression levels of *Cx26, Cx29, Cx30, Cx30.2, Cx30.3, Cx31, Cx31.1*, Cx32, *Cx33*, Cx37, *Cx43, Cx45, Cx46, Cx50, Cx57, Oct3/4, Rex1, Cdx2, Gata4*, and *β-actin* were analyzed by qRT-PCR in SYBR Green PCR Master Mix (Applied Biosystems) using a StepOnePlus^TM^ Real-Time PCR System (Applied Biosystems). Primer sets and product sizes of respective target RNAs are listed in supplemental table (Table S2). The amount of target mRNA was normalized to the amount of *β-actin* mRNA.

#### Separation of the cell membrane and the cytosol fractions of EB3

EB3 cells growing in each culture dish were washed with PBS. Then 2 mL HEPES buffer solution (20 mM HEPES, 250 mM sucrose, 2 mM EDTA, pH 7.4) was added to the dish to collect cells with a scraper. Adding 3 mL HEPES buffer solution, the cell suspension was sonicated (UR-20P, TOMY SEICO Co. Ltd., Tokyo, Japan) on ice and centrifuged at 2,000 g for 10 min. The supernatant was collected and centrifuged at 12,000 g for 20 min. Then the supernatant was centrifuged again at 180,000 g for 90 min. The supernatant was collected as the cytosol fraction. The precipitate was suspended in a RIPA buffer (25 mM Tris-HCl, 150 mM NaCl, 1% NP-40, 1% sodium deoxycholate, 0.1% SDS, pH 7.6; Thermo Fisher Scientific, Waltham, MA, USA) and centrifuged at 15,000 g for 20 min. The supernatant was collected as the cell membrane fraction.

#### Western analysis of Cx30.3 protein

Protein sample solutions were prepared from EB3 cells, their cell membrane fraction, their cytosol fraction, Cx30.3 overexpressing EB3 cells, the kidney and the spleen of a C57BL/6 N mouse. Mouse kidney was positive control, while mouse spleen was the negative control, respectively. The protein concentration was determined using a Pierce^®^BCA^TM^ Protein Assay kit (Thermo Fisher Scientific).

A sample solution containing 30–50 μg protein was mixed with a 1/6 volume of buffer solution containing 0.375 M Tris-HCl (pH 6.8), 93 μg/mL DTT, 0.12 g/mL SDS, 0.6 mL/mL glycerol, and 0.6 mg/mL bromophenol blue. The mixed solution was then heated at 95 °C for 5 min and proteins were separated by SDS-PAGE at 30 mA.

Blotting onto a PVDF membrane was conducted at 300 mA for 3 h at 4 °C. The PVDF membrane was then immersed in 5% skim milk dissolved in Tris-buffered saline (25 mM Tris-HCl, pH 7.5, 0.15 M NaCl) containing 0.1% Tween 20 (TBS-T) for 1 h at 4 °C with gentle shaking. After overnight incubation at 4 °C with rabbit anti-mouse Cx30.3 polyclonal antibody (40–0900, Thermo Fisher Scientific) in 5% skim milk/TBS-T solution with shaking at 40 rpm, the PVDF membrane was washed three times with TBS-T and incubated with anti-rabbit immunoglobulin conjugated to alkaline phosphatase (Promega) for 1 h at 25 °C, with shaking at 40 rpm. The PVDF membrane was subsequently incubated with Western Blue Stabilized Substrate for alkaline phosphatase (Promega) for 5 min at 25 °C.

To re-probe for β-actin, the PVDF membrane was incubated with mouse anti-β-actin antibody conjugated to alkaline phosphatase (Santa Cruz Biotechnology, Inc., Dallas, TX, USA) at 25 °C for 2 h, and then stained for alkaline phosphatase as described above. The re-probe for α1 Na^+^ -K^+^ ATPase, a cell membrane marker was conducted with mouse monoclonal anti-α1 Na^+^ -K^+^ ATPase antibody conjugated to alkaline phosphatase (ab7671, Abcam) in the same manner. Stained image was quantified using Image J (http://imagej.nih.gov/ij/).

#### Fluorescent microscopy

EGFP and DsRed were introduced to visualize overexpressing Cx proteins. PKH26 (Sigma-Aldrich) was used to stain membrane structure. Those fluorescent images were observed with a confocal laser scanning microscope (LSM510, Carl Zeiss Co., Ltd., Jena, Germany) and also with an all-in-one fluorescence microscope (BZ-X700, Keyence Co., Osaka, Japan).

#### Immunostaining of endogenous Cx30.3 and Cx43 proteins

EB3 cells were cultured in ESM for 24 h. The medium was removed and the cells were washed twice with PBS. The cells were treated with 4% paraformaldehyde on ice for 15 min for fixation. After incubation 3 times in 10 mM glycine-PBS for 5 min, the blocking treatment was conducted by the incubation in 2% gelatin-PBS for 20 min followed by the incubation 3 times with PBS-glycine, and the incubation with 0.1% BSA-PBS for 5 min. And then the cells were reacted with the primary antibody at 37 °C for 40 min in 1% BSA-PBS containing a rabbit anti-mouse Cx30.3 polyclonal antibody (1:500 dilution) (40–0900, Thermo Fisher Scientific) or a rabbit anti-Cx43 polyclonal antibody (1:2000 dilution) (ab11370, Abcam, Cambridge, UK). After the reaction, the blocking treatment was conducted 6 times with 0.1% BSA-PBS for 5 min. Then the cells were reacted with the second antibody at 37 °C for 40 min in 1% BSA-PBS containing an anti-rabbit immunoglobulin antibody labelled with Alexa Fluor 488 (1:300 dilution) (Invitrogen). Finally the cells were washed 6 times with 0.1% BSA-PBS for 5 min.

#### Cx30.3 overexpressing cell line

An overexpression vector for Cx30.3 was constructed by inserting the Cx30.3 gene into pCAG-gene-IRES-EGFP (donated by Dr. H. Niwa, RIKEN). The vector product (4 μg/250 μL GMEM) and lipofectamine 2000 solution (10 μL/250 μL GMEM) were gently mixed and incubated for 20 min at 25 °C. The mixture was then added to culture dishes of 90% confluent EB3 cells and incubated for 4 h. The cells were subsequently cultured at 37 °C for 24 h and then G418 was added to the medium at 1.5 μg/mL to select cultures over 7 d. After washing with PBS, an appropriate colony was picked with a micro-pipette, transferred to a 10 μL trypsin solution and incubated for 3 min at 25 °C. ESM (100 μL) was then added to the solution and the cells were re-suspended. The cell suspension was transferred into a well of a 48-well plate to which 500 μL ESM/well was added beforehand.

Another set of overexpression vectors for fusion proteins, Cx30.3-EGFP, Cx30.3-DsRed, and Cx43-EGFP, was prepared by inserting a connexin gene into pCMV-gene-EGFP or pCMV-gene-DsRed for use in the investigation of their intracellular localization. The vector products were linearized by treating with *ApaLI* and introduced into EB3 cells by electroporation using a GENE PULSER II (Nippon Bio-Rad, Tokyo, Japan). Transfected cells were selected as outlined above.

#### Cx30.3 and Cx43 co-overexpressing cell line

Overexpression vectors for Cx30.3 and Cx43 were simultaneously introduced into EB3 cells. pCMV-Cx30.3-DsRed (2 μg/250 μL GMEM) and pCMV-Cx43-EGFP (2 μg/250 μL GMEM) were prepared respectively and then mixed with lipofectamine 2000 (10 μL/250 μL GMEM). The mixture was stirred gently and stood still for 20 min at 25 °C. The mixture was then added to culture dishes of 90% confluent EB3 cells and incubated for 4 h. The cells were subsequently cultured at 37 °C for 24 h and then G418 was added to the medium at 1.5 μg/mL to select cultures over 7 d.

#### Immunostaining of SSEA1

SSEA1 is a pluripotent state marker for mouse cells and was used to confirm that EB3 cells in colonies could maintain the pluripotent state even after dissociation into single-cells. EB3 cells were cultured in a dish with a diameter of 35 mm containing LIF(+) medium. After the culture at 37 °C for 3 d, the medium was removed and the cells were washed with PBS. Four mL PBS-EDTA solution was added to the dish and incubated for 5 min. Then cells were separated from the dish bottom using a cell scraper, and transferred into a 15 mL centrifugation tube. After the centrifugation at 1500 rpm × 5 min at 4 °C, the precipitate was suspended in 600 μL PBS. Cells (10^6^) in 2 mL of 3% BSA/PBS were added to a centrifugation tube and then centrifuged again as above. The precipitate was suspended in 100 μL 3% BSA/PBS and rested for 10 min at 25 °C for blocking. To this suspension, mouse anti-SSEA1 antibody (sc-21702, Santa Cruz Biotechnology) was added and incubated for 1 h at 25 °C. The supernatant was removed by centrifugation at 1500 rpm × 5 min at 4 °C and then donkey anti-mouse IgG labelled with Alexa488 (A21202, Thermo Fisher Scientific) was added and cells were incubated for 30 min at 25 °C. After centrifugation at 1500 rpm × 5 min at 4 °C, the precipitate was suspended in 0.5–1.0 mL PBS.

#### Flow cytometry

A cell suspension was centrifuged at 1500 rpm × 5 min at 4 °C and the precipitate was re-suspended in 0.5–1.0 mL PBS and then filtered with a cell strainer (pore size 35 μm). The cell suspension was analyzed with a flow cytometer (FACSAria II, BD Biosciences, San Jose, CA, USA). The fluorescence of Alexa488 was detected at 515–545 nm by excitation at 488 nm.

Based on the flow cytogram indicated by the histogram of the number of cells versus the fluorescence intensity, the fractions of control cells emitting auto-fluorescence, SSEA1 negative cells, and SSEA1 positive cells were collected and then applied to qRT-PCR as described above.

#### Analysis of mRNA half-time

EB3 cells were cultured in LIF(+) medium in 6-well plates. The initial cell density was adjusted at 1 × 10^5^ cells/well. After 24 h, the medium was replaced by a fresh LIF(+) medium containing actinomycin D (Wako Pure Chemical Industries, Ltd., Osaka, Japan) at 5 μg/mL to inhibit transcription. After prescribed times, the medium was removed and the cells were collected by means of ISOGEN II. RNA extraction and qRT-PCR were conducted in the same way as described above.

#### Statistics

Test sample preparation of mRNA or protein was conducted by one test sample per one dish. In most cases, three test samples were analyzed per one analytical item. One test sample was analyzed twice and the average of the two results was recorded as the value for the test sample. At critical points, two-sided Student’s *t*-test was conducted to estimate statistical significance.

## Additional Information

**How to cite this article**: Saito, M. *et al*. Connexin30.3 is expressed in mouse embryonic stem cells and is responsive to leukemia inhibitory factor. *Sci. Rep.* **7**, 42403; doi: 10.1038/srep42403 (2017).

**Publisher's note:** Springer Nature remains neutral with regard to jurisdictional claims in published maps and institutional affiliations.

## Supplementary Material

Supplementary Dataset 1
