# Supplementary material for: Connexin30.3 is expressed in mouse embryonic stem cells and is responsive to leukemia inhibitory factor
Source: Sci Rep. 2017 Feb 13;7:42403. doi: 10.1038/srep42403 (PMC5304323; doi:10.1038/srep42403)
Supplement: Supplementary Dataset 1 [file srep42403-s1.doc]

(resubmitted to Scientific Reports)

Category: Articles

Connexin30.3 is expressed in mouse embryonic stem cells and is responsive to leukemia inhibitory factor

Mikako Saito^*^, Yuma Asai, Keiichi Imai, Shoya Hiratoko, Kento Tanaka

Department of Biotechnology and Life Science, Tokyo University of Agriculture and Technology, 2-24-16, Naka-cho, Koganei, Tokyo 184-8588, Japan.

^*^Corresponding author:

Mikako Saito

Department of Biotechnology and Life Science, Tokyo University of Agriculture and Technology, 2-24-16, Naka-cho, Koganei, Tokyo 184-8588, Japan.

TEL; +81-42-388-7400, FAX; +81-42-387-1503, E-mail; mikako@cc.tuat.ac.jp

**Supplementary figure and table legends**

Figure S1. Cx30.3-EGFP localization image data captured by time-lapse measurement for 3480 s.

Image data at 0, 960, and 1440 s (indicated by red squares) were selected for Fig. 4c. Scale bar: 10 μm.

Table S1. Primers used for RT-PCR.

Table S2. Primers used for qRT-PCR.

Fig. S1

Table S1. Primers used for RT-PCR.

Table S2. Primers used for qRT-PCR.
